# Aqueous Solubility of Organic Compounds for Flow Battery Applications: Symmetry and Counter Ion Design to Avoid Low-Solubility Polymorphs

**DOI:** 10.3390/molecules26051203

**Published:** 2021-02-24

**Authors:** Sergio Navarro Garcia, Xian Yang, Laura Bereczki, Dénes Kónya

**Affiliations:** 1 Central Research Institute of Natural Sciences, Magyar Tudósok körútja 2, 1117 Budapest, Hungary; sergio.navarro@ttk.hu (S.N.G.); nagyne.bereczki.laura@ttk.mta.hu (L.B.); 2 JenaBatteries GmbH, Otto-Schott-Straße 15, 07745 Jena, Germany; xian.yang@jenabatteries.de

**Keywords:** organic redox flow battery, hydroquinone, solubility, solubility prediction, polymorph, Ostwald’s rule

## Abstract

Flow batteries can play an important role as energy storage media in future electricity grids. Organic compounds, based on abundant elements, are appealing alternatives as redox couples for redox flow batteries. The straightforward scalability, the independence of material sources, and the potentially attractive price motivate researchers to investigate this technological area. Four different benzyl-morpholino hydroquinone derivatives were synthesized as potential redox active species. Compounds bearing central symmetry were shown to be about an order of magnitude less soluble in water than isomers without central symmetry. Counter ions also affected solubility. Perchlorate, chlorate, sulfate and phosphate anions were investigated as counter ions. The formations of different polymorphs was observed, showing that their solubility is not a function of their structure. The kinetics of the transformation can give misleading solubility values according to Ostwald’s rule. The unpredictability of both the kinetics and the thermodynamics of the formation of polymorphs is a danger for new organic compounds designed for flow battery applications.

## 1. Introduction

As renewable energy penetration increases, the importance of energy storage grows accordingly to compensate its intermittency [1]. Redox flow batteries are one possible answer to the emerging need of storing energy. In these batteries, energy is stored in the form of the chemical energy of two different chemical redox pairs. The greatest advantage of flow batteries is that their rated power and stored energy/capacity can be adjusted independently, which makes them the ideal candidate for grid applications.

Traditionally, most flow batteries were based on the different oxidation states of metals as electrolytes [2]. The first semi-organic system was published in 2014 [3], followed by the first all-organic one in 2014 [4]. Since that time, several other chemistries have been published [5,6,7]. A redox flow battery must fulfil several criteria to be commercially successful; such as low cost, safe operation, and sustainable production scalability. When water is used as a solvent, safe operation is guaranteed. The organic electrolyte makes the scale up independent from locally available resources. However, to ensure a low storage cost: cheaply executable synthesis routes, chemically stable and highly water-soluble compounds are needed. The concentration of the electrolyte is important for several reasons. In grid applications, energy density is not a critical issue as in the case of electro-mobility, but a higher electrolyte concentration increases the final energy density as well as the achievable current densities, leading to better usage of the reactor unit as a whole and reducing capital and maintenance costs. Finally, high electrolyte concentration increases the overall cycle efficiency by cycling less solvent (ballast) through the pumps.

Unfortunately, organic syntheses are not always straightforward. They are often the result of many trial and error experiments conducted by synthetic chemists. Synthesizing a target compound with the required analytical specifications is often a tedious and timely process. Due to the difficulties in the syntheses and the importance of the solubility factor, it would be desirable to be able to predict the solubility of the target compound before its synthesis, so as to avoid all problems that can arise in synthesic procedures.

Pharma researchers have been predicting different physicochemical properties of organic compounds for a long time. Although some properties like logP and logD values can be predicted with acceptable accuracy [8], solubility remains a difficult task. The story of ritonavir [9] andthe USD 250 million loss of Abbott on the issue in 1998 when it was finally closed [10], highlighted how desperately the pharma industry needs a useful prediction method for the aqueous solubility of organic compounds. A project titled “The solubility challenge” was launched in 2008 by scientists from Cambridge University and AstraZeneca [11], challenging theoretical chemists in their paper: “Can You predict solubilities of 32 molecules using a Database of 100 Reliable Measurements?”. There were 99 researchers who participated in the challenge and sent their predictions. In 2009 the results were analysed in the following paper: “Findings of the challenge to predict aqueous solubility” [12]. Its conclusions were that no method could reliably predict the solubility of many organic compounds. The partition coefficient and distribution coefficient are the ratio of concentrations of a compound in a mixture of two immiscible solvents in equilibrium. The partition coefficient describes the ratio of uncharged species, while the distribution coefficient describes all species of the given compound. This explains why the latter is often pH dependent. Their logarithmic values are logP and logD, which are commonly used physicochemical parameters to express “greasiness” among pharma chemists. Their calculated values, which are referred as clogP and clogD are quite predictable. Why is it more difficult to predict solubility than the logP or logD values? The devil is in the details, so it is necessary to investigate what energies occur during dissolution. Firstly, the solute–solute and the solvent–solvent interactions should break up, and then the solute–solvent interactions are formed. The solubility is determined by the sum of these three energies. The latter two are reliably calculable, thus logP and logD values, which use these two energies, are also predictable. However, the question remains as to what can be said about the energies of solute–solute interactions. Fortunately, this area has also been researched. Researchers of crystal structure predictions (CSPs) are continually challenging themselves. The last CSP challenge in 2016 was the sixth, where five unpublished organic compounds’ crystal structures were predicted. Although the computational methods and capacities have advanced considerably, they were still not accurate and reliable enough to give predictions about the energies in solid phase structures to serve as a basis for solubility predictions. One of the most difficult tasks is to predict polymorphs. Polymorphs are different crystal structures from the same given compound. Polymorph formation can be influenced by the solvent, the rate of the crystal growth, temperature, pressure, etc., indicating that the crystal structure is not only a function of a given compound, but also depends on many other parameters. Ritonavir has two main polymorphs (the two which caused the problem), but an additional three were found during the investigation of the issue. Different polymorphs have different solubility values—five in the case of the structure of ritonavir [13].

Additionally, even the kinetics of polymorph formation work against flow battery researchers. According to Ostwald’s rule, it is often the polymorph with the higher solubility (less-packed crystal structure) that is discovered sooner, and the polymorphs with lower solubility (more ordered crystal structure) are discovered later [14]. This means that a newly isolated compound’s solubility can change over time after its first isolation. Unfortunately, this is most likely to happen in an unfavorable direction.

In this work, two pairs of hydroquinone derivatives were synthesized, whose chemical structures were confirmed by the characterization of NMR and MS, followed by the solubility measurement in four kinds of acids via gravimetric and NMR methods. We show how the appearance of new polymorphs can cause unexpectedly huge changes in solubility, what methods can be applied to avoid the formation of these densely packed crystals that will lead to low solubility, and how to obtain structures with better solubility.

## 2. Results

Our research investigated different quinone derivatives as potential electroactive compounds for all organic flow battery applications. The electrochemistry of these derivatives will be discussed separately, in another article. Quinone is a good lead from an electrochemistry perspective. Its redox potential is sufficiently high, its kinetics are fast, and the quinone/hydroquinone reactant pair represents a two-electron reaction where all participants are closed-shell compounds. However, neither its stability nor its solubility are high enough for practical applications, making its derivatization inevitable. The Mannich reaction is a straightforward option for its derivatization to build a C–C bond onto a phenolic hydroxyl-containing aromatic ring. It is also important to protect the ortho position of the phenolic hydroxyl as much as possible, due to its easily tautomerizable hydrogens and its radical electron in the radical-ion formation during the electrode reaction.

**Figure 1:**
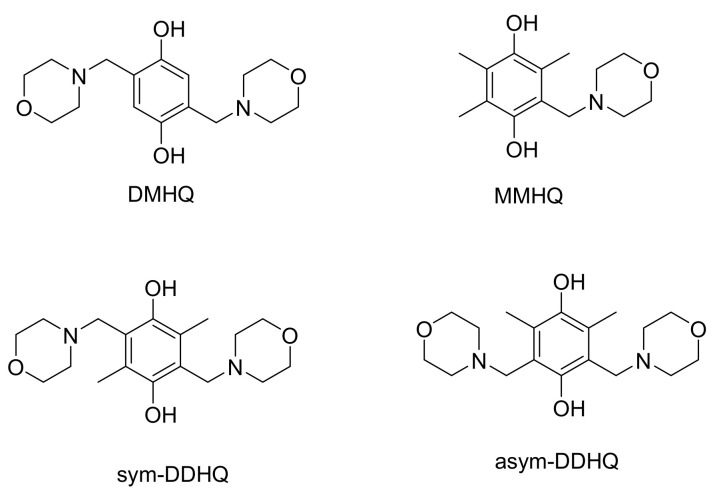
Synthesized Mannich products. IUPAC names of the compounds: DMHQ: 2,5-bis[(morpholin-4-yl)methyl]benzene-1,4-diol; MMHQ: 2,3-dimethyl-5-[(morpholin-4-yl)methyl]benzene-1,4-diol; sym-DDHQ: 2,5-dimethyl-3,6-bis[(morpholin-4-yl)methyl]benzene-1,4-diol; asym-DDHQ: 2,6-dimethyl-3,5-bis[(morpholin-4-yl)methyl]benzene-1,4-diol.

**Figure 2:**
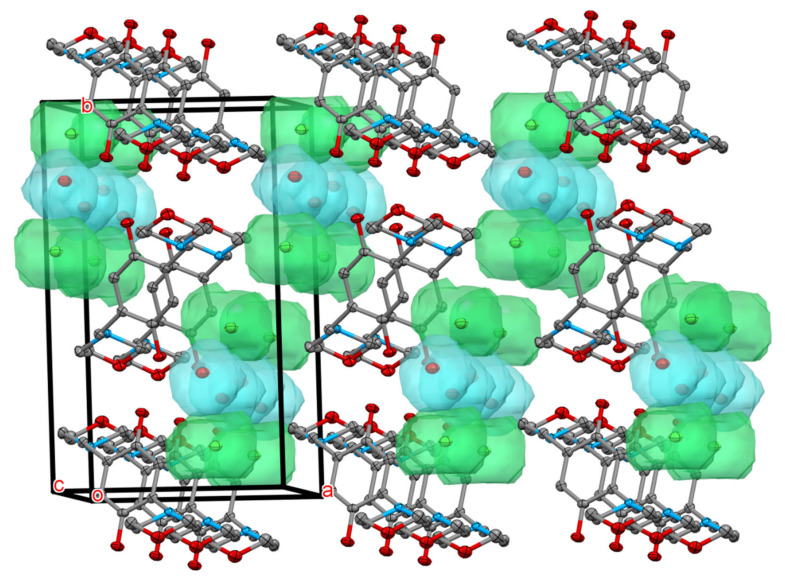
Crystal structure of the hydrochloric salt of DMHQ. Hydrogens are omitted for clarity. Green balls are chloride ions, blue balls are water molecules, and the light green and blue spots show the space available for them in the crystal.

Four hydroquinone derivatives were synthesised using the Mannich reaction (Figure 1) [15]. DMHQ was originally reported in 1964 with only simple structural characterizations and its chemical properties were not covered [16]. Its NMR spectrum was acquired in deuterated chloroform in later research in our laboratory. The aqueous solubility of DMHQ in 1 M sulfuric acid was surprisingly good, reaching a concentration of 1.7 M as measured by gravimetry. Unexpectedly, its solubility dropped below 0.1 M one year later. It was supposed that the chemical structure of DMHQ had been changed. Our supposition that the structure had changed was initially reinforced by the fact that the stored compound was so poorly soluble that the spectrum was no longer measurable in deuterated chloroform. However, the spectrum acquired in DMSO-d5 later showed that the product had not been changed chemically. Instead, the compound’s chemical integrity remained intact; the formation of a new polymorph led to its low solubility. The hydrochloric acid salt of DMHQ was proven to be a good candidate for X-ray analysis, resulting in a crystal structure where this polymorph was quite densely packed (Figure 2, Table 1).

**Figure 3:**
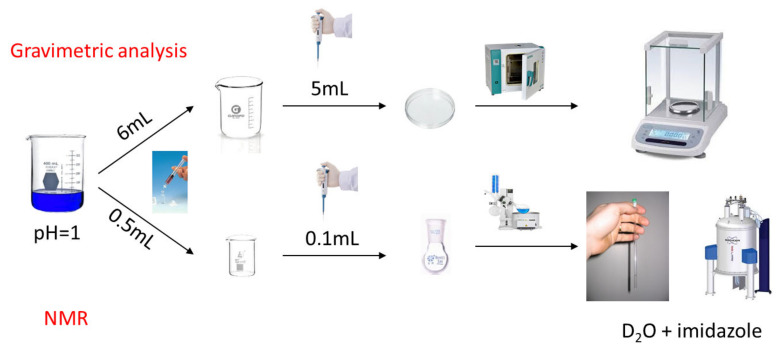
Flow chart of the gravimetric and NMR methods used for determining solubility.

Figure 3 illustrates the two methods used to determine solubility. The first method was gravimetry which required a higher amount of analyte to reduce any error arising from possible co-crystalized compounds. This method is easy to realize without the need for high-tech equipment, and its result is also a reference for the other method performed by NMR spectroscopy. The NMR method required only a small quantity of the analyte and was more accurate as no co-crystalized compounds were counted in its homogeneous solution phase.

The NMR spectra used to determine the solubility were recorded with a known amount of imidazole as the internal standard. After processing the NMR spectra, the solubility results of the four hydroquinone derivatives are summarized in Table 2.

As supposed from the X-ray structure, anions with different charge and space needs could alter the crystal structure and therefore its solubility. Chloride and perchlorate, which are commonly used anions in organic salt crystals, were poorly soluble, whereas the final solubility of sulfate crystals was strongly dependent on the structure of the cation pair. Phosphate had good solubility in all cases. This demonstrates that sulfuric acid is a better choice for an organic flow battery application because of its higher conductivity, better availability, and cheaper price. As seen in Table 2, central symmetry had a detrimental effect on solubility in sulfuric acid in the cases of DMHQ and sym-DDHQ. This is based on the supposition that these compounds create better packed solids which results in higher lattice energy, the energy of the solute–solute interactions, which means lower solubility. However, when the central symmetry was broken and crystal formation was hindered, higher solubility was found (MMHQ, asym-DDHQ).

## 3. Discussion

**Figure 4:**
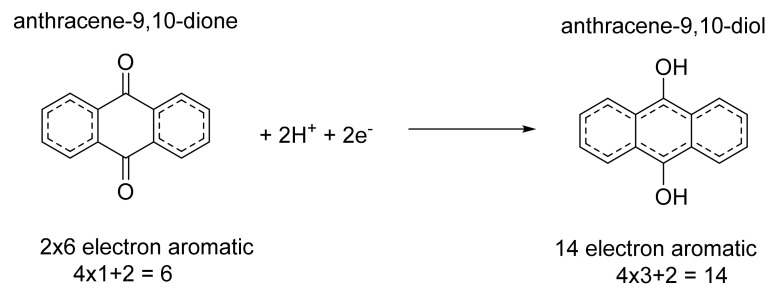
Aromaticity of the anthraquinone/dihydro-anthraquinone redox pair according to Hückel’s rule.

The results of this study clearly indicate that organic compounds at higher concentrations can behave unexpectedly in aqueous conditions. Similar experiences have been found by researchers working in the field of organic flow batteries. Anthraquinone disulfonic acid (AQDS) is a popular choice due to its chemical stability, which comes from the fact that both the oxidized form (2 × 6 electrons) and the reduced form (1 × 14 electron) fulfil the 4n+2 conjugated ring electron in Hückel’s rule, so both are aromatics (Figure 4). It can be seen that the anthraquinone/dihydro-anthraquinone pairs were the smallest system with this characteristic (together with its same molecular weight isomer: the phenantrene-9,10-dione).

**Figure 5:**
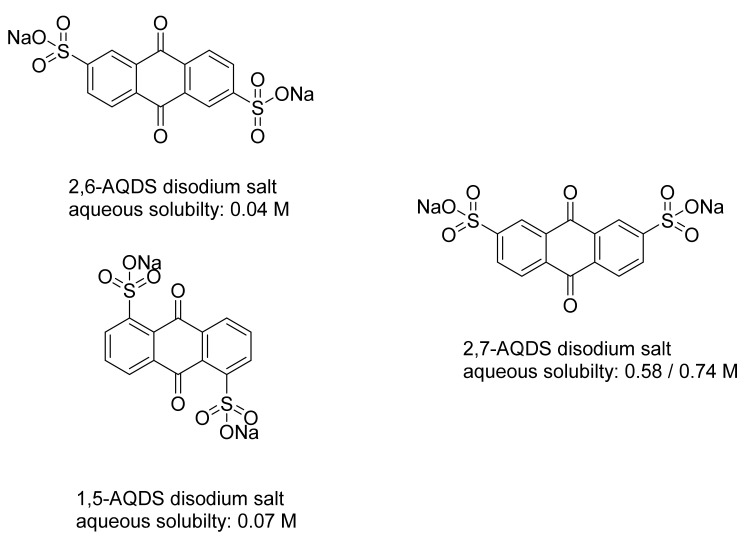
Solubility difference between 1,5-, 2,6-, and 2,7-AQDS disodium salts. The 1,5- and 2,6-AQDS derivatives have central symmetry while the 2,7-AQDS does not. Their difference in solubility is about an order of magnitude. The solubility of 2,6-AQDS disodium salt was determined using the NMR method with imidazole as the internal standard.

The solubility differences between different isomers of AQDS is well known [17]. The 9,10-dioxo-9,10-dihydroanthracene-2,6-disulfonic acid disodium salt (2,6-AQDSNa_2_) is poorly soluble in water, with an aqueous solubility below 0.1 M. The same is true for 9,10-dioxo-9,10-dihydroanthracene-1,5-disulfonic acid disodium salt (1,5-AQDSNa_2_), which also has central symmetry. It has an aqueous solubility of 0.07 M. The asymmetric analogue of 2,6-AQDS and 1,5-AQDS without central symmetry is the 9,10-dioxo-9,10-dihydroanthracene-2,7-disulfonic acid (2,7-AQDS). Its disodium salt’s solubility is 0.58 M/0.74 M; the difference is about an order of magnitude higher compared to those of the 2,6- and 1,5-AQDS disodium salts (Figure 5).

**Figure 6:**
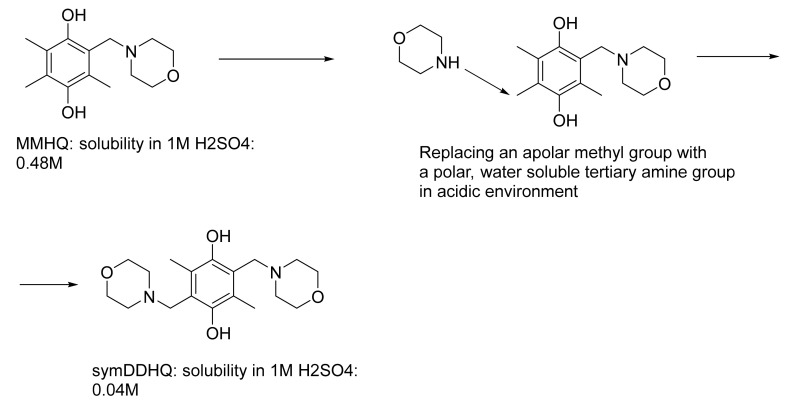
This virtual reaction shows how strongly the molecular symmetry can influence the solubility.

Analogous to the 1,5- and 2,6- vs. 2,7-AQDS derivatives, the solubility results of the four compounds presented in this work show a similar phenomenon regarding the effect of the molecular symmetry and the applied counter ion on the corresponding aqueous solubility, as listed in Table 2. The example in Figure 6 further demonstrates the importance of taking into consideration the molecular symmetry when designing novel redox-active organic molecules targeting high solubility. The aqueous solubility of MMHQ in 1 M sulfuric acid was 0.48 M. When replacing the apolar methyl group with the polar tertiary amine group, enhanced solubility is expected in acidic conditions. However, the solubility of sym-DDHQ decreased by an order of magnitude to 0.06 M. Even though the freshly obtained product had a solubility of 0.24 M in 1 M sulfuric acid, a more stable polymorph formed about a year after the first measurement showed the lower 0.06 M value. In this case, the molecular symmetry apparently dominated the impact on the aqueous solubility. As a comparison, the asym-DDHQ without central symmetry had a solubility of 0.44 M in 1 M sulfuric acid (Table 1).

Therefore, it can be concluded that structural asymmetry can have a positive effect on molecular solubility, even by an order of magnitude. On the other hand, it is clear that counter ions have an important role in solubility as well. This is because the charge and size of the counter ion in the crystal determine the solubility by influencing the lattice energies of the different crystal structures.

Practically, the AQDS derivatives used as negolytes in flow battery applications will probably be a mixture of isomers because the sulfonation of anthraquinone cannot be directed to give only one product. Separation of these isomers on an industrial scale is impractical due to the time and resources required to find totally new synthetic approaches which could create too many risks.

Consequently, targeting quinone derivatives without molecular central symmetry is encouraged in order to achieve the best solubility. If there is any doubt over whether the intrinsic solubility can be found, then the potentiometric cycling for the polymorph creation ([PC]^2^) method is a useful tool to find the corresponding polymorph in their order of formation [18]. It is highly recommended that [PC]^2^ experiments are run before upscaling a potential candidate to avoid possible surprises with lower-solubility polymorphs.

## 4. Materials and Methods

### 4.1. Nuclear Magnetic Resonance Spectroscopy (NMR)

^1^H NMR spectra were recorded in DMSO-d6 or CDCl_3_ solution at room temperature, on a Varian Unity Inova 500 spectrometer (Bruker Corp. Oxford, UK) (500 and 125 MHz for ^1^H NMR and APT-NMR spectra, respectively), with the residual solvent signal as the lock and imidazole as the internal standard (only for solubility determination). Chemical shifts (δ) and coupling constants (J) are given in ppm and Hz, respectively.

### 4.2. Liquid Chromatography-Mass Spectroscopy (LC-MS)

HPLC-MS measurements were performed using a Shimadzu LCMS-2020 (Shimadzu Corp. Kyoto, Japan) device equipped with a Reprospher (Altmann Analytik Corp. München, Germany) 100 C18 (5 μm; 100 mm× 3 mm) column and a positive/negative double ion source (DUIS) with a quadrupole MS analyzer in the range of 50–1000 *m/z*. The samples were eluted with gradient elutions, using eluent A (0.1% formic acid in water) and eluent B (0.1% formic acid in acetonitrile). The flow rate was set to 1.5 mL/min. The initial condition was 5% eluent B, followed by a linear gradient to 100% eluent B by 1.5 min; from 1.5 to 4.0 min, 100% eluent B was retained; and from 4 to 4.5 min, it returned by a linear gradient to 5% eluent B, which was retained from 4.5 to 5 min. The column temperature was kept at room temperature, and the injection volume was 1–10 μL. The purity of the compounds was assessed by HPLC with UV detection at 215 and 254 nm; all starting compounds were known, purchased, or synthetically feasible, and >95% pure.

### 4.3. X-ray Crystallography

A colorless single crystal of DMHQ.HCl was mounted on a loop with paratone oil. Intensity data were collected on a Raxis Rapid II diffractometer (graphite monochromator; Cu-Kα radiation, λ = 1.54178 Å) at 140 K. The structures were solved by charge-flipping methods (and subsequent difference syntheses) and then anisotropic full-matrix least-squares refinement on *F*^2^ for all non-hydrogen atoms was performed. The hydrogen atomic positions could be located on difference Fourier maps. Hydrogen atomic positions were calculated from assumed geometries. Hydrogen atoms were included in the structure factor calculations, but they were not refined. The isotropic displacement parameters of the hydrogen atoms were approximated from the *U*(eq) value of the atom they were bonded to. Molecular graphics were made by the Mercury software.

CCDC 2058624 contains the Supplementary Crystallographic Data for this paper.

### 4.4. Synthesis

All hydroquinone derivatives were synthesized via a one-step Mannich type condensation from the corresponding hydroquinone.

General method for the Mannich reaction (DMHQ, MMHQ, sym-DDHQ): To a homogeneous solution of morpholine (3 eq.) and paraformaldehyde (3 eq.) in 20 mL of isopropyl alcohol, a solution of the corresponding hydroquinone (10 g, 1 eq.) in 50 mL of isopropyl alcohol was added. The mixed solution was refluxed for 1.5 h and then concentrated to give a white crystalline raw product. The product was washed with two 50 mL portions of diethyl-ether and dried naturally.

In the case of asym-DDHQ, a different procedure was used: a homogeneous solution of morpholine (19 mL, 4 eq.) and paraformaldehyde (6.5 g, 4 eq.) in 50 mL of toluene and 50 mL of 2-(2-ethoxyethoxy) ethanol was heated at 110 °C and the distillate was removed. Then 2,6-dimethylbenzoquinone (10 g, 1 eq.) was slowly added. The mixed solution was heated for 20 h at 145 °C and then concentrated. Remaining solvents were distilled and the crude product was purified by filtering through a pad of silica gel using hexane/ethyl acetate (*v/v* = 3:7) with two drops of triethylamine as eluent.

Sym-DDHQ needed a previous hydrogenation step to synthesize the corresponding hydroquinone derivative (2,5-dimethylhydroquinone), since its starting material is available commercially only in the quinone form (p-xyloquinone): To a solution of p-xyloquinone (10 g, 1 eq.) in 100 mL of methanol, 5% Pd/C (0.1 eq.) was added in an autoclave and the autoclave was closed. The mixture was kept at 40 °C and three charges of 7.5 bar of H_2_ were introduced. After the total consumption of H_2_, the mixture was filtered through a pad of celite to remove Pd/C residues and the filtrate was concentrated. Recrystallization of the filtrate in methanol gave 2,5-dimethylhydroquinone.

^1^H nuclear magnetic resonance (NMR), high-performance liquid chromatography (HPLC), and mass spectroscopy (MS) were performed to confirm the structure of targeted molecules:

**DMHQ** (2,5-bis[(morpholin-4-yl)methyl]benzene-1,4-diol): ^1^H NMR (500 MHz, CDCl_3_): δ 6.49 (2H, s), 3.74 (8H, s), 3.65 (4H, s), 2.56 (8H, s); ^1^H NMR (500 MHz, DMSO): δ 9.33 (2H, s), 6.53 (2H, s) 3.58 (8H, t, *J* = 5 Hz), 3.47 (4H, s), 2.40 (8H, s); HPLC rt = 0.985 min; MS (*m/z*) = 309.

**MMHQ** (2,3-dimethyl-5-[(morpholin-4-yl)methyl]benzene-1,4-diol): ^1^H NMR (500 MHz, CDCl_3_): δ 3.74 (4H, s), 3.70 (2H, s), 2.56 (4H, s), 2.16 (9H, s); HPLC rt = 1.468 min; MS (*m/z*) = 252.

**sym-DDHQ** (2,5-dimethyl-3,6-bis[(morpholin-4-yl)methyl]benzene-1,4-diol): ^1^H NMR (500 MHz, DMSO): δ 10.5 (2H, s), 3.63(4 H, s), 3.59 (8H, t, *J* = 5 Hz), 2.45 (8H, s), 2.04 (6H, s); HPLC rt = 0.994 min; MS (*m/z*) = 337.

**asym-DDHQ** (2,6-dimethyl-3,5-bis[(morpholin-4-yl)methyl]benzene-1,4-diol): ^1^H NMR (500 MHz, CDCl_3_): δ 3.74 (8H, t, *J* = 5 Hz), 3.70 (4 H, s), 2.59 (8H, s), 2.24 (6H, s); HPLC rt = 0.919 min; MS (*m/z*) = 337.

## 5. Conclusions

This article shows that polymorph formation is a real danger in compound design for organic flow batteries, as it can drastically reduce the aqueous solubility of the target compound by forming a more densely packed crystal. The biggest issue is that the precise and reliable prediction of aqueous solubility is still not possible regardless of polymorph formation. The timescale of the appearance of the polymorph with lower solubility is unpredictable as well. In the case of forming salts, changing the counter ion is a possible option if other conditions permit. Our general advice is that it is better to avoid the central symmetry of the targeted molecules in order to decrease the probability of the formation of a stable polymorph that will undesirably lower the solubility. This statement is true for AQDS derivatives as well—another important compound group in flow battery chemistry.

## Figures and Tables

**Table 1:** Crystal data of the hydrochloric salt of DMDQ.

Compound	DMDQ.2HCl
Empirical formula	C16 H30 Cl2 N2 O6
Formula weight	417.32
Temperature	140(2)
Radiation and wavelength	Cu-Kα, λ = 1.54187 Å
Crystal system	monoclinic
Space group	P 21/c
Unit cell dimensions	a = 9.2629(2) Å
	b = 15.4919(3) Å
	c = 7.4246(2) Å
	α = 90°
	β = 110.973(8)°
	γ = 90°
Volume	994.84(6) Å^3^
Z	2
Density (calculated)	1.393 Mg/m^3^
Absorption coefficient, μ	3.238 mm^−1^
F(000)	444
Crystal color	colorless
Crystal description	prism
Crystal size	0.72 × 0.48 × 0.38 (mm)
Absorption correction	numerical
Max. and min. transmission	0.5390.766
θ−range for data collection	5.114 ≤ θ ≤ 68.172°
Index ranges	−11 ≤ h ≤ 10; −18 ≤ k ≤ 18; −8 ≤ l ≤ 7
Reflections collected	17,903
Completeness to 2θ	0.972
Independent reflections	1763 [R(int) = 0.0332]
Reflections I > 2σ(I)	1733
Refinement method	full-matrix least-squares on F^2^
Data/restraints/parameters	1763/0/134
Goodness-of-fit on F^2^	1.184
Final R indices [I > 2σ(I)]	R1 = 0.0308, wR2 = 0.0787
R indices (all data)	R1 = 0.0317, wR2 = 0.0793
Max. and mean shift/esd	0.000; 0.000
Largest diff. peak and hole	0.249; −0.244 e.Å^−3^

**Table 2:** Solubilities of different quinone derivatives in 1 M acids.

Compound	Cl^−^	ClO_4_^−^	SO_4_^2−^	PO_3_^3−^
MMHQ	0.07 ^1^	0.02	0.48	0.48
DMHQ	0.03 ^1^	0.01	0.08 ^2^	0.53
sym-DDHQ	-	-	0.06 ^3^	0.40
asym-DDHQ	-	-	0.44	0.55

Notes: ^1^ These two compounds’ gravimetric analysis was used to validate the NMR method; ^2^ First isolated polymorph showed 1.7 M solubility by the gravimetry method; ^3^ First polymorph showed 0.24 M solubility by the NMR method.

## Data Availability

Not applicable.

## Supplementary Materials

The following are available online, crystallographic data.

## Sample Availability

Research samples of hydroquinone derivatives are available by e-mailing to Denes Konya (konya.denes@ttk.mta.hu).
