# Supplementary material for: Aqueous Solubility of Organic Compounds for Flow Battery Applications: Symmetry and Counter Ion Design to Avoid Low-Solubility Polymorphs
Source: Molecules. 2021 Feb 24;26(5):1203. doi: 10.3390/molecules26051203 (PMC7956567; doi:10.3390/molecules26051203)

checkCIF/PLATON report

Structure factors have been supplied for datablock(s) shelx

THIS REPORT IS FOR GUIDANCE ONLY. IF USED AS PART OF A REVIEW PROCEDURE FOR PUBLICATION, IT SHOULD NOT REPLACE THE EXPERTISE OF AN EXPERIENCED CRYSTALLOGRAPHIC REFEREE.

No syntax errors found. CIF dictionary Interpreting this report

Datablock: shelx

```
Bond precision:  C-C = 0.0020 A         Wavelength=1.54187

Cell:            a=9.2629(2)     b=15.4919(3)        c=7.4246(2)
                 alpha=90        beta=110.973(8)     gamma=90
Temperature:     140 K
```

	Calculated	Reported
Volume	994.84(7)	994.84(6)
Space group	P 21/c	P 21/c
Hall group	-P 2ybc	-P 2ybc
Moiety formula	C16 H26 N2 O4, 2(Cl), 2(H2 O)	C16 H26 N2 O4 2+, Cl2 2-, 2(H2O)
Sum formula	C16 H30 Cl2 N2 O6	C16 H30 Cl2 N2 O6
Mr	417.32	417.32
Dx,g cm-3	1.393	1.393
Z	2	2
Mu (mm-1)	3.238	3.238
F000	444.0	444.0
F000’	446.71	
h,k,lmax	11,18,8	11,18,8
Nref	1817	1763
Tmin,Tmax	0.194,0.292	0.539,0.766
Tmin’	0.078	

```
Correction method= # Reported T Limits: Tmin=0.539 Tmax=0.766
AbsCorr = NUMERICAL

Data completeness= 0.970          Theta(max)= 68.172

R(reflections)= 0.0308( 1733)     wR2(reflections)= 0.0793( 1763)

S = 1.184                  Npar= 134
```

```
The following ALERTS were generated. Each ALERT has the format
       test-name_ALERT_alert-type_alert-level.
Click on the hyperlinks for more details of the test.
```

Alert level C

```
PLAT029_ALERT_3_C _diffrn_measured_fraction_theta_full value Low .      0.972 Why?
PLAT906_ALERT_3_C Large K Value in the Analysis of Variance ......      2.390 Check
PLAT911_ALERT_3_C Missing FCF Refl Between Thmin & STh/L=    0.600         51 Report
PLAT934_ALERT_3_C Number of (Iobs-Icalc)/Sigma(W) > 10 Outliers ..          1 Check
```

Alert level G

```
PLAT042_ALERT_1_G Calc. and Reported MoietyFormula Strings  Differ     Please Check
PLAT063_ALERT_4_G Crystal Size Possibly too Large for Beam Size ..       0.72 mm
PLAT398_ALERT_2_G Deviating  C-O-C    Angle From 120 for O1             109.6 Degree
PLAT909_ALERT_3_G Percentage of I>2sig(I) Data at Theta(Max) Still        96% Note
PLAT912_ALERT_4_G Missing # of FCF Reflections Above STh/L=  0.600          3 Note
PLAT978_ALERT_2_G Number C-C Bonds with Positive Residual Density.          4 Info
```

```
   0 ALERT level A = Most likely a serious problem - resolve or explain
   0 ALERT level B = A potentially serious problem, consider carefully
   4 ALERT level C = Check. Ensure it is not caused by an omission or oversight
   6 ALERT level G = General information/check it is not something unexpected

   1 ALERT type 1 CIF construction/syntax error, inconsistent or missing data
   2 ALERT type 2 Indicator that the structure model may be wrong or deficient
   5 ALERT type 3 Indicator that the structure quality may be low
   2 ALERT type 4 Improvement, methodology, query or suggestion
   0 ALERT type 5 Informative message, check
```

Validation response form

Please find below a validation response form (VRF) that can be filled in and pasted into your CIF.

```
# start Validation Reply Form
_vrf_PLAT029_shelx
;
PROBLEM: _diffrn_measured_fraction_theta_full value Low .      0.972 Why?
RESPONSE: ...
;
_vrf_PLAT906_shelx
;
PROBLEM: Large K Value in the Analysis of Variance ......      2.390 Check
RESPONSE: ...
;
_vrf_PLAT911_shelx
;
PROBLEM: Missing FCF Refl Between Thmin & STh/L=    0.600         51 Report
RESPONSE: ...
;
_vrf_PLAT934_shelx
;
PROBLEM: Number of (Iobs-Icalc)/Sigma(W) > 10 Outliers ..          1 Check
RESPONSE: ...
;
# end Validation Reply Form
```

It is advisable to attempt to resolve as many as possible of the alerts in all categories. Often the minor alerts point to easily fixed oversights, errors and omissions in your CIF or refinement strategy, so attention to these fine details can be worthwhile. In order to resolve some of the more serious problems it may be necessary to carry out additional measurements or structure refinements. However, the purpose of your study may justify the reported deviations and the more serious of these should normally be commented upon in the discussion or experimental section of a paper or in the "special_details" fields of the CIF. checkCIF was carefully designed to identify outliers and unusual parameters, but every test has its limitations and alerts that are not important in a particular case may appear. Conversely, the absence of alerts does not guarantee there are no aspects of the results needing attention. It is up to the individual to critically assess their own results and, if necessary, seek expert advice.

Publication of your CIF in IUCr journals

A basic structural check has been run on your CIF. These basic checks will be run on all CIFs submitted for publication in IUCr journals (*Acta Crystallographica*, *Journal of Applied Crystallography*, *Journal of Synchrotron Radiation*); however, if you intend to submit to *Acta Crystallographica Section C* or *E* or *IUCrData*, you should make sure that full publication checks are run on the final version of your CIF prior to submission.

Publication of your CIF in other journals

Please refer to the *Notes for Authors* of the relevant journal for any special instructions relating to CIF submission.

PLATON version of 05/12/2020; check.def file version of 05/12/2020

Datablock shelx - ellipsoid plot

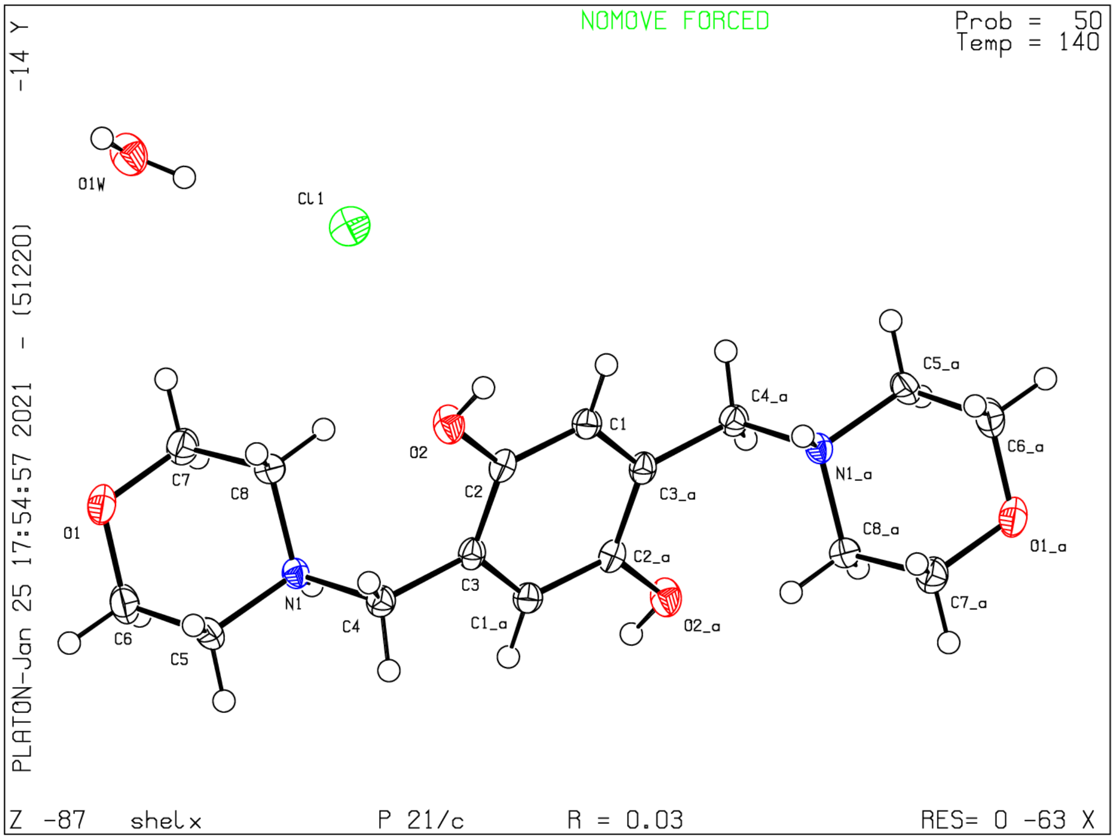

Supplement: Supplementary file 1 [file molecules-26-01203-s001.zip › molecules-1109261-SM/checkcif2058624.pdf]
